# The impact of digitalization and organizational changes on older workers' insecurity in the finance sector in Sweden and Czechia

**DOI:** 10.3389/fsoc.2026.1835265

**Published:** 2026-06-25

**Authors:** Alena Křížková, Clary Krekula, Martina Rašticová, Marie Heřmanová, Jennie Sjödin

**Affiliations:** 1 Department of Gender and Sociology, Institute of Sociology, Czech Academy of Sciences, Prague, Czechia; 2 Department of Social Work, Linnaeus University, Kalmar, Sweden; 3 Faculty of Business and Economics, Mendel University, Brno, Czechia; 4 Department of Cultural Anthropology and Ethnology, Uppsala University, Uppsala, Sweden

**Keywords:** digitalization, insecurity, older workers, organizational changes, population aging

## Abstract

**Introduction:**

There are still knowledge gaps relating to the interaction between digitalization, organizational changes, and the aging of the workforce. This study examines the impact of digitalization on older workers in the financial sector in Sweden and the Czechia. The experience of digitalization by older workers in different countries and labor market sectors, and its fostering of insecurity and precarity through technostress, feelings of inefficacy and uncertain futures, is understudied.

**Methods:**

Through interviews with men and women aged 50–64 who are employed in financial institutions in the Czechia and Sweden, the research explores organizational changes, including outsourcing and agile management, and their impact on the experiences of older workers at work.

**Results:**

The findings reveal key differences in ongoing changes between the two countries, such as the prominence of agile management in Sweden and the absence of outsourcing in the Czechia. Despite these variations, digitalization narratives are remarkably similar, reflecting an international emphasis on efficiency and competitiveness. The study illustrates that digitalization can create precarious working conditions for older employees, including technostress, feelings of ineffectiveness and insecurity, and uncertainty about the future.

**Discussion:**

The results emphasize the need for temporal analyzes and consideration of national conditions when understanding the multifaceted consequences of digitalization for the older workforce. Overall, the paper highlights the similarities and differences in organizational opportunities and challenges within and across national labor market contexts, providing valuable contributions to the ongoing academic debate on aging and digitalization in the workplace.

## 1 Introduction

European societies are strongly influenced by the ongoing “digital revolution” (Charles et al., 2022). Digitalization is not confined to offices or IT firms; however, prognosis shows that about 22% of workforce activities across the EU (equivalent to 53 million jobs) could be automated by 2030 (McKinsey Global Institute, 2020). Digitalization provides both opportunities and challenges as work could become more efficient, healthy, and creative, but also more insecure, stressful, and difficult (Charles et al., 2022). Simultaneously, European labor markets experience a “demographic revolution” since the share of 50+ workers is larger than ever (European Commission, 2023). While population aging and digitalization are two current megatrends, the impact of their intersection in the European labor markets is not sufficiently examined. Extension of working lives (EWL) is the generally adopted response to aging in the developed welfare states (Kuitto and Helmdag, 2021; Phillipson, 2019). How is EWL and organizational changes, which are adopted in digitalizing workplaces, experienced by different age groups and in different welfare state and organizational contexts should be explored.

It is still not sufficiently clear whether digitalization primarily hurts or helps the position of older workers as the impact is not direct but is shaped by a composition of factors (Piasna, 2024) and contextual variations, such as sectors (Křížková et al., 2025) and country institutional variation (Lloyd and Payne, 2023). For example, on one hand, the digital platforms and the gig economy can provide flexible employment opportunities for older workers, where older workers can choose hours and intensity of work that fit their needs (Piasna and Drahokoupil, 2017). On the other hand, automation, digitalization and AI may threaten low-skilled jobs, leading to job insecurity among older workers (Piasna, 2024). Moreover, a wide digital skills gap between youth and older generations (Pak, 2021) and ageism (Scoppetta et al., 2024) can hinder the participation of the older population of workers in a digitalized economy. This uncertainty makes it crucial to understand the interplay between aging and digitalization (Fernández-Ardèvol and Grenier, 2022). Recently, Eurofound (2025) suggests that EWL requires actions concerning collective bargaining and social dialogue and not only legal measures. Phillipson (2019) calls for a re-thinking of the notion of the “older worker” and for more intensive research of the links between population aging and policies extending working lives on one hand, and technological development and how it changes the workplace on the other.

The financial sector has emerged as a leader in the adoption of digitalization (European Commission, 2020). However, the impact of digitalization on older workers remains a subject of ongoing academic debate, revealing knowledge gaps regarding the organizational changes and insecurities accelerated by digitalization, as well as the experiences of older workers. This paper aims to fill the research gap and respond to calls for more in-depth sectoral analysis specifically focused on the under researched workplace level of how EWL policies and organizational changes including digitalization are experienced by older workers themselves.

One aspect of organizational change in the financial sector is the prevalence of fusions and acquisitions, resulting in a consolidation of strong players in the market and the emergence of smaller, more innovative companies that are adaptable to rapid changes (Thalassinos, 2008). While mergers and acquisitions have primarily affected traditional banks and financial institutions, the working conditions in smaller banks often exhibit a start-up culture (Gimpel et al., 2018). Studies have indicated that older workers may encounter challenges in adapting to new technologies implemented during mergers and acquisitions or within start-ups (Kooij et al., 2013). However, further research is necessary to understand the specific organizational factors that contribute to these difficulties.

Both these organizational changes and digitalization can lead to job insecurities for older workers, including concerns about job displacement, technological obsolescence, and skills gaps (Eurofound, 2025). Research is needed in how digital technologies are adopted and how this adoption changes organizational structure and processes and the situation of older workers facing at the same time EWL in different country contexts.

To address these gaps, this study explores organizational changes, such as outsourcing and agile management, as well as job insecurities, including technostress experienced by older employees in the finance sector in Sweden and Czechia. This paper is based on qualitative interviews conducted with women and men, aged 50–64, employed in financial institutions in Sweden and Czechia.

By focusing on the financial sector, which is at the forefront of digitalization, and comparing two very different country contexts, this paper contributes findings on how the contrasting trends of digitalization and the EWL intersect in the experiences of older workers. Digitalization and organizational restructuring, which are often associated with a young workforce (Salento, 2018), may lead to an increase in the insecurity experienced by older workers, who are simultaneously encouraged to extend their working lives. While there are significant differences in the organizational adoption of new technologies and management practices, with Czechia lagging behind Sweden, digitalization and agile management related restructuring are experienced similarly by older workers in both countries, causing insecurity, ageism, and precarization.

## 2 Literature review—Older employees in the financial sector

Since the 2010s, the financial sector, which is the focus of this article, has undergone considerable changes. A significant change consisted of fusions and acquisitions leading to a limited number of strong actors in the financial market. This development took place concurrently with the establishment of smaller and often more innovative companies, whose primary strength was their adaptability to rapid changes (Thalassinos, 2008). While employees in traditional banks and financial institutions were mostly affected by the fusions, the working conditions in the new smaller banks were characterized by a so-called start-up culture (Gimpel et al., 2018). These changes have contributed to increased precarity for employees as acquisitions, among other things, came with large scale downsizing (Rogovsky and International Labour Office, 2005).

A significant factor boosting the organizational change in the financial sector is outsourcing, even though as a practical strategic tool it has appeared for a long time (Deavers, 1997). The broad definition of outsourcing is that it obtains activities from outside of the organization. Over the last few years outsourcing-banking operations has gathered momentum, and the nature of the outsourced tasks has evolved (Dibbern and Hirschheim, 2020).

These trends have contributed to different; often customer driven management models (Dong et al., 2024). The agile management method marked by the agile software development and lean management practices, is recurrently described as standard in the bank and financial sector (Dong et al., 2024). This management approach was originally mainly a product design strategy that was meant to be adaptive, as opposed to anticipatory (Dikert et al., 2016). While there are not many studies analyzing the impact of agile management introduction on individual employees, Theobald et al. (2020) argue that it has some positive impact on organizational practices, for example greater inclusiveness and transparency.

Digitalization is a “global megatrend that is fundamentally changing existing value chains across industries and public sectors” (Collin et al., 2015, p. 29). Terms such as “mobile Apps, Big Data, Machine-to-Machine, Internet of Things, Industrial Internet, and Industry 4.0” are used to describe this phenomenon (Collin et al., 2015, p. 29). Examples of digitalization, or digital transformation when discussing the actual change-process (Porfírio et al., 2024), can be seen in the media (streaming services like Netflix and Hulu, transforming the way people consume entertainment), banking (digital transformation has enabled online banking, mobile banking apps, and contactless payments), telecom (Telecom companies like Verizon and AT&T have embraced 5G technology and cloud computing to improve connectivity and offer innovative services), and insurance industries (AI-powered claims processing, reducing the time and effort required to settle claims) as pioneering sectors that are in the middle of large-scale digital transformation (Collin et al., 2015). For some years now, research focuses on the effect of digitalization of the banking sector on employment (Meena, 2019), work organization and the quality of work (Porfírio et al., 2024). Studies in this field often focus on the perceived autonomy of employees, feelings of loss of control, flexibilization of work and technostress or digital stress (Gimpel et al., 2018). Still, there is a lack of knowledge about the consequences of digitalized work organizations for older employees.

In the finance sector, a variety of studies have explored working conditions during ongoing changes. Examples include explorations of organizational commitment among employees in the banking sector (Khan et al., 2021), factors affecting employees' organizational commitment in banks (Nguyen and Tran, 2021), and job satisfaction in banking industries (Dhamija et al., 2019). A prominent issue that has emerged is technostress, defined as an inability to cope with technology, leading to distress (Brod, 1984). The consequences span various disorders in workers and organizational losses, such as fatigue, dissatisfaction, anxiety, and reduced productivity. Individual factors like age influence the perception and coping with technostress. While older workers may experience less technostress, they might find technological complexity more challenging (Marchiori et al., 2019), interruptions more stressful (Tams and Hill, 2017), and organizational social media more distracting (Brooks et al., 2017).

Studies of such issues in other occupational sectors highlight that subjective insecurity increases with age; education reduces job insecurity, as do wages, except at very high wage levels; having a temporary contract contributes to insecurity (Muñoz de Bustillo and De Pedraza, 2010). Job-related insecurity is mostly related to the job content, e.g., complex or monotonous work, too many responsibilities, conflicting or ambiguous demands (Hulshof et al., 2020), working conditions, employment conditions, e.g., temporary employment (Sora et al., 2019), work-family conflict (Nauman et al., 2020), and job performance (Pilipiec, 2020). Digitalization has brought a new wave of uncertainty, particularly for older workers who may lack the technological expertise to adapt to the changing landscape (Degryse, 2016), negotiating from scratch of post-retirement employment contract can lead to uncertainty, unfairness (Wainwright et al., 2019) and inequality in job satisfaction (Bolli and Pusterla, 2022). Workplace-level research is scarce and pointing to ageism and ongoing practices marked by negative view of older workers not able to adapt to organizational and technological change (Le Feuvre and Rougier, 2023; Křížková et al., 2025). Despite these investigations, the unique perspective of how ongoing organizational changes affect older employees' experience of uncertainty remains an underexplored area. Existing in-depth qualitative research is represented mostly by single-country studies and therefore this paper is contributing with the comparative perspective.

## 3 Theoretical framing—Ageism, precarization, EWL, and digitalization

The analysis of ongoing organizational changes within the finance sector in the two countries is based on the premise that collective understandings of key concepts and processes are not created through “diffusion.” Instead, we follow the understanding that this occurs through “translations” within work organizations and related international collaborative bodies, which in some cases results in a common understanding of central concepts, while in other cases it opens up varying outcomes in different local contexts (Czarniawska and Joerges, 1996). Against this background, our analysis aims to identify both similarities and differences in the current organizational discourses between the two countries and to problematize these outcomes. In our analysis, the concepts of ageism and precarity are central to understanding the consequences of organizational changes in both Swedish and Czech contexts.

In academic literature, we can find several overlapping definitions of ageism (Eppler-Hattab et al., 2024). Iversen et al. (2009) define it as negative or positive stereotypes, prejudice and/or discrimination against aging people because of their chronological age. Perceived age discrimination influences workers in many age groups with a negative impact on job satisfaction, commitment, and engagement (Macdonald and Levy, 2016; Lössbroek et al., 2021). Older workers might be affected by the digital divide, and internalized ageism can occur when older workers self-stereotype as less able to learn digital technologies (Hampel and Kunze, 2023).

Precarity has until recently been conceptualized as a social condition linked to poverty (Lazar and Sanchez, 2019). According to Kreshpaj et al. (2020), precarity can be defined as an “atypical form of employment of lower quality with potential adverse internal consequences” (p. 429). This can manifest, for example, short-term and part-term contracts, self-employment, and contract work, which often leads to instability. Lazar and Sanchez (2019), however, point out that the essential character of formal employment has in late capitalism changed in a way that it erodes the certainty and fixity previously associated with long-term employment contracts. This means that precarity is now experienced also by people who have employment contracts and stable wages—but their position is so vulnerable and characterized by so much uncertainty that it needs to be characterized as precarious. Similarly, Lorey (2020) argues that we should focus on the process of *precarization* as opposed to the *state of precarity* and points out that this process is much more complex than insecure jobs and instability. This approach also sheds light on how the processes of precarization particularly impacts older workers as it is connected with extending working life and related policies.

In contemporary labor markets, older workers are positioned at the intersection of digitalization, organizational restructuring and policies that promote EWL. This configuration can intensify job insecurity and broader forms of precarity. Although digitalization is frequently discussed in terms of enhancing productivity and employability (Kornelakis et al., 2022; Peng et al., 2017) or macro-level risks (Arntz et al., 2019), these viewpoints often neglect the organizational contexts in which technological change is implemented (Howcroft and Taylor, 2023). The impact of digital technologies depends on organizational strategies and practices (Howcroft and Taylor, 2023); therefore, more attention should be paid to how workplace processes mediate their effect on different groups. Within organizational processes, age is one of the key axess of inequality and older workers are disproportionately exposed to the risks of technological substitution and exclusion (Moen et al., 2017), while digital competence is commonly perceived as a characteristic of youth (Salento, 2018). Organizational regimes of inequality reproduce these hierarchies through differential access to training, opportunities and recognition, which are often reinforced by stereotypes about the adaptability and skills of older workers (Lössbroek et al., 2021). At the same time, EWL policies operate within a broader neoliberal framework that individualizes responsibility for continued employment and adaptation to change (Lain et al., 2022) frequently without adequate institutional or organizational support.

## 4 Contexts, material, and method

The article analyses the situation in Czechia and Sweden. These countries have been affected by the above-described changes to different degrees. In terms of organizational structure, the Czech finance sector has undergone the agile management transformation process in recent years (mostly 2019 onwards) and the impact was significant both on the organizational hierarchy and structure and on individual employees. In Sweden, the first bank underwent the agile management transformation in 2012 and most others between 2016 and 2018 (Khan et al., 2021). Significant differences can also be found in the varying timeframes and levels of digitalization. Banks in Sweden have worked with automation processes since the 1970s. In Czechia, the process started 20 years later, with the formation of banking institutions operating in the transforming market environment in the late 1990s and early 2000s. The varying degrees of digitalization in both countries are also reflected in their DESI index ranking, where Sweden is consistently ranked as the frontrunner (4th place in DESI index ranking in 2022) and Czechia remains in the second half of EU member states ranking (19th place in DESI index ranking in 2022) (European Commission, 2022).

The composition of workers in the finance sector regarding age is similar in both countries; the largest age group among employees is 40–49 years (see Czech Statistical Office ^1^, Statistics Sweden^2^). The retirement age in Sweden is flexible between 63 and 69; 65 in Czechia.

### 4.1 Sample, research methodology, and analytical process

The sample from both countries consists of qualitative semi-structured interviews (Magaldi and Berler, 2020) with older workers and trade union and HR representatives in the banking sector. The Czech sample of older workers includes 37 qualitative interviews with 25 women and 12 men working in the banking sector. Their age was between 50 and 67, with an average of 56.9 for women and 58.1 for men. The employees were in positions of personal bankers, local branch managers, administrators, controllers, and cash center specialists. The average tenure within one financial company was 21 years. The interviews were carried out in eight different banking and insurance companies all over Czechia. The Swedish sample consists of 38 interviews, 20 women and 18 men, ages between 49 and 62. The average age of the informants was 57.4 for the women and 56.7 for the men. The informants were representing positions such as local bank branch managers, project leaders in main administrative centers, trade union representatives, financial advisors to businesses or private persons, and customer support workers. Most informants had been employed in the sector for several decades, many even 30 years or more at the same company. The interviews were carried out in 10 different companies with locations all over Sweden. The sample was complemented by 10 expert interviews with trade union and HR representatives in the Czech and three in the Swedish banking sector. We used the snowball sampling recruitment strategy focused on employees over 50 years of age and on trade union and HR representatives in the banking sector in both countries. We focused on workers aged 50+ because this group is at a particularly important stage of late working life, where experiences of digitalization, organizational change, and employment insecurity intersect with age-related expectations, accumulated work trajectories and concerns about future labor market participation.

Most of the empirical data were collected between 2019 and 2020, with four expert interviews conducted in 2023 in Czechia. The interviews were conducted at respondents' workplaces and ranged in duration from 1 to 2 h. Interviews with bank employees focused on narratives of personal and work history, current working conditions, perceived effects of aging at work, organizational attitudes toward older workers, interactions with health, family, and care responsibilities, anticipation of and preparation for the transition to retirement, retirement plans, financial circumstances, and personal opinions on EWL (see the Interview guide for employees in Supplementary file 1). Expert interviews focused on age awareness within the organization, organizational attitudes toward older workers, existing and/or planned EWL measures, ongoing organizational changes, and personal views on aging at work (see the Interview guide for experts in Supplementary file 2). The interview collection was part of a wider research project focused on experiences of 50+ employees with the EWL in three sectors (banking, public transport, and nursing). The focus on experiences with digitalization and organizational changes emerged during the analysis of the interviews. The interviews were recorded, fully transcribed, anonymized, and coded using ATLAS.ti. For the purposes of this article, the analysis focused on working conditions, specifically employees' experiences with organizational changes, including digitalization, and their perception on both individual and organizational level.

The transcribed interview material has been used for a thematic analysis (Braun et al., 2019). The empirical analysis was an interpretative process that was carried out in several steps. Passages in the transcribed material were analyzed thematically (Guest et al., 2012), illustrating the reasoning connected to the meanings of age, working conditions, changes in the organization of work, and future work prospects. The research team worked in two country teams, in the national languages, and adopted an intensive analytical collaboration through continuous comparative discussion of coding and findings, instead of conducting more technical coding comparison checks with the use of the software. After the first set of close readings of the interviews, focusing on parts concerning digitalization and organizational changes, the author team developed a basic coding structure. During online meetings, we collaborated closely in the coding process for all the interviews. The Czech and Swedish teams met online every 2–3 weeks for 6 months to discuss the analysis and decide on further steps in developing the coding structure. This coding structure was developed continuously through close collaboration and based on an agreement that followed the close coding, collection, sorting and classification of translated quotations from the interviews. Following several discussions and repeated processes of reading, coding and common discussions about older workers' experiences connected to digitalization, the research team decided to divide its findings into two basic themes: (1) organizational changes and (2) experiences of insecurity. During subsequent meetings, the research team generated subcodes that were analytically saturated by quotations from the interviews. The subcodes and their saturation were systematically compared between the two countries through shared discussions, and the coding structure was refined (see the final Coding structure in the Supplementary file 3). The teams reflected extensively on the findings within the thematic area and the comparative approach. For instance, outsourcing was identified as a significant theme in the Swedish interviews, yet it received limited discussion in the Czech interviews. Therefore, during the analytical process, the research team decided to conduct four more interviews with HR and other banking experts specifically about outsourcing and other organizational changes. This enabled us to achieve saturation of the data on organizational changes.

The following results part is structured based on these two themes and subthemes and on systematic comparison of the data from Czechia and Sweden.

## 5 Results

### 5.1 Organizational changes: international processes and national interpretations

When the informants from Czechia and Sweden give accounts of how they experience the main organizational changes, three themes emerge: digitalization, out-sourcing processes and agile management transformation. Below we illustrate the similarities and differences in these narratives in the two countries.

#### 5.1.1 Different phases of established change processes—Agile management and out-sourcing

The empirical data illustrate differences in the experiences of organizational changes, as can be seen in the narratives about agile management. In the Czech material, there are recurring statements on agile leadership, as exemplified in the following extract:

*The client can always intervene and control the process and say, nah, I don't want that, scratch that. So, with these shorter time periods in the agile approach, we should be like, more effective, but that's a problem, we're still learning to live with it*. (45-year-old female trade union representative)

This description illustrates how agile management prioritizing the needs of the customers (Dong et al., 2024) is present but not yet lodged in established routines at this nationally leading bank. In the Swedish material, however, agile management seems to be more of an accepted practice, as shown in statements such as ”*One of our core values is to be agile and that is what we feel we are*” and “*Because that's how we work in the bank, agile*.” This is also clear in statements saying that challenges and advantages have already been identified, such as in:

“*As people talk about ‘this agile' now, where you can be very mobile in different groups and so on.” and “That is the advantage of working agile, that all unit work, like together, you decide across borders as well. Which makes us pull in the same direction.”* (61-year-old woman, Sweden)

A further difference revealed lies in the way the informants from the two countries talk about outsourcing. Outsourcing does not appear as a significant restructuring process in the studied Czech banks as illustrated by the fact that older employees in Czechia did not talk about outsourcing when describing organizational changes in their banks. Accounts of the HRs show, that outsourcing is a rather marginal process with no impact on the structure or operation mode of the banks in Czechia: “*Mostly, only administrative or support services are outsourced, such as receptionists or cleaning staff, and also IT services*” and “*The outsourced entities are small or individual specialists who temporarily provide specific service or develop specific product or system to the bank*.” or “*No core services provided by the bank are outsourced.”*

In the Swedish case, descriptions of outsourcing and its consequences frequently occur when informants talk about central organizational changes, as in the following statement by a 53-year-old woman bank employee:

*The trend is to outsource to cheap countries. And there's a lot to Poland and there's a lot to India. [ ] And it has suffered inflation, it has been glorified and there's inflation in “My god, see how much money we can save by moving to Poland. What other processes can we move there?” And then it's done in absurdum*.

The differences illustrated above in the Czech and Swedish material can be understood as reflecting that the banks in these countries are in different phases of the two processes discussed. While the first bank in Sweden underwent the agile management transformation in 2012 and most others between 2016 and 2018 (Khan et al., 2021), this approach was introduced in Czechia mostly in 2019 and onwards (Gillior, 2022). Corresponding differences apply to outsourcing. While the Swedish banks rely on the typical model of outsourcing to countries and sectors with cheaper workforce, the Czech banks practice internal outsourcing, so called insourcing, where tasks are transferred to members of the same organization or unit.

#### 5.1.2 Fewer people, more work

Our data illustrate similarities in how the informants in both countries understand the digitalization of banking processes as an organizational change. Digitalization is introduced in banking with the aim of simplifying and reducing work; however, our data reveal two processes that show that this is rather a myth.

The first process we identified in the data is the workload that does not decrease. While some positions are eliminated, and people are cut off by the digitalization of the banking processes, there are new tasks, such as more administration. Many operations are digitalized, as well as they are kept on paper, and more and more supporting documents must be put into digital databases. The accounts of employees in both countries show this unfulfilled promise of digitalization. One Czech older bank employee explains: “*They are constantly promising less and less paperwork and less internal regulation, yet there is more and more, and each unit defends its paperwork and its methodology*.” Another one sums up: “*There are fewer and fewer people, but not less work*.” Similarly, a Swedish bank employee is explaining how the time saved by digitalization when some tasks are eliminated is eaten up by new tasks, such as increasing administration: “*Well, digitalization simplifies things. But I think that we all experience that all the added rules... eat up the time saved through the digitalization*.” In other words, with digitalization, the workload does not decrease.

The accounts in both countries also show that with digitalization the work pace increased. For example, an older employee in Sweden explains: “*Tempo. Answering or doing things are accelerating all the time*.” A Czech trade union leader observes the change in banking in the last decade: “*The pressure on performance and pace, increasing the pace of work, work changed a lot in banks over the last ten years; it is completely different than in the past*.” Taken together, in both countries, digitalization is understood as causing a *decrease* in the workforce, but in parallel an *increase* in the workload and work pace.

Previous research on the impact of digitalization on work focused more on changes in the structure of the labor market and elimination of certain jobs (Frey and Osborne, 2017) and changes to forms of employment relations (Piasna and Drahokoupil, 2017) and less on the content of jobs. Digitalization is expected to improve efficiency and productivity of the labor process (Kornelakis et al., 2022) as well as to assign new functions and competence requirements to the remaining jobs (Ilsøe, 2017). Our results provide further perspective on these processes by illustrating that new, mainly administrative tasks have been added to the workload of those who remain, resulting in an overall increase in work. In contrast to the expectations of less and more efficient work, we find that for those who remain, there is higher workload and higher work tempo.

The second process causing rather more work for bank employees, which we identified in both countries, is the responsibility of employees for their own training. As digitalization means a constant change in the work process and the IT systems, each new digital banking application, operating system, and administrative regulation requires a learning process. An employee with tenure of over 20 years in a big bank in Czechia reflects the change in the training process over time: “*Earlier when a new application was introduced it was trained, today you just get information about new applications via Skype.”* The following accounts of a Swedish and Czech employee, respectively, illustrate that employees in both countries are expected to manage their training themselves. “*A lot has happened for us at the office, new knowledge that one must take in and all the digital training one must go through. Of course, it's tougher*” and “*There is very little training in banking or software here*.” Our data show that constantly seeking their training, training themselves, and looking for available support became an added work in digitalizing banks. Taken together, with digitalization there is more work conducted by fewer people faster, and with the added work of learning new digital processes on their own.

Above we have illustrated that organizational processes such as the implementing of agile management and outsourcing vary in the two countries. We have argued that this can be seen as an expression of national variations regarding temporality of the development of the banking sector, where and when, for example, different wage costs are relevant for the implementation of outsourcing. We have also shown that the digitalization process is ongoing in both countries and is presented with the goal of reducing and simplifying work. However, contrary to these assumptions, our results show that this has not been achieved in practice, as people and positions are cut off, while new tasks emerge.

The presence of both organizational differences and similarities, which has been illustrated above, can be understood as an expression that the discussed processes at least partially follow different organizational interpretations (see Czarniawska and Joerges, 1996). While the implementation of agile management and outsourcing is influenced by factors that are largely shaped nationally, digitalization can be understood as an international process where technologies and the valuation of them quickly move between national and organizational contexts.

### 5.2 Digitalization as a source of stress and insecurity

As an organizational change on global scale, the rapidly digitalizing banking sector represents a challenge for the working conditions of older workers. When focusing on everyday experiences of older bank employees with digitalization we find three themes that are similarly experienced in the accounts of our informants in Sweden and Czechia: (1) increased stress, (2) they do not feel needed and wanted within the organization due to their age, and (3) insecurity about the future. Below we discuss these themes.

#### 5.2.1 Experiences of stress

When the informants in the two countries talk about the consequences of digitization in their own workplaces, stories about various dimensions of stress emerge. One aspect is related to the numerous and rapidly changing software, in which the interviewees work. An example of this is given by a 59-year-old woman from Czechia who states: “*For me, it was another stress that the work is done in many systems. Switching between systems, and my biggest fear was that I wouldn't know what was where*.” Along the same lines, a 55-year-old Swedish man speaks about how this also creates a dependence on IT support that ultimately results in a feeling of being alone when things go wrong: “*You should manage that yourself. There is IT support but there is so much you need to manage yourself*.”

The experiences of stress are also related to the fact that digitalization creates an increased pace while reducing the workforce, which has been discussed above. A 55-year-old bank employee in Sweden describes it as follows: “*Improved efficiency has not led to slower tempo. Instead, digitization and improved efficiency have led to the same tempo, only that fewer people do the same thing... more things*.” A Czech female 47-year-old HR manager provides another example of this when she talks about standing meetings: “*Everybody stands up and says what they did, what they‘re going to do that day, and what they're going to do tomorrow, and I have to say, it's very stressful for some people*.”

In the Swedish material, there are also statements about how digitization brings about new regulations and increased administration, which is expressed by a 57-year-old man as follows: “*Well, digitalization in itself simplifies things. But I think that we all experience that all the added rules [ ] eat up the time saved through the digitalization of something else*.”

Overall, the above results illustrate that the expectations of digitization reducing stress appear simplified in relation to the more complex changes that digitization brings for the informants in the two countries. The experiences of stress described above can be understood as expressions of different forms of technostress (Nimrod, 2018), where the rapid development of information and communication technology with constant change (Ayyagari et al., 2011) and the anxiety and sense of ineffectiveness that its use can also entail are central.

#### 5.2.2 Older workers don't feel needed and wanted

The empirical material from the two countries shows that among the older financial employees there is an experience of not having or not being expected to have the skills that are in demand in a work organization characterized by digitization. This is expressed in statements such as these from the Swedish informants “*I usually jokingly say that I'm the last generation that didn't grow up with a computer*” and “*The banks need people who know IT, and I haven't worked with that. I don't have that education. I have finance at the bottom and... banking.”* Czech informants express similar reflections, as in the following account: “*The knowledge and the know-how of the technology. After all, we're from a generation, where we didn't have much room to develop that*.”

The informants also state that these changes with constant new programs are taking place at an increasingly rapid pace. A male financial employee in Sweden expresses it as the changes were previously more limited and that “*It was a little new but not much. That has sort of accelerated. So, from one year to another, if you think about the last year, an incredible amount has happened for us in the office*.” A female employee in Czechia conveys the same thought: “*The development of technology goes so fast, so I wonder what will happen next*.”

These experiences of having the wrong and outdated skills contribute to a feeling of not being needed and wanted. The following two quotes illustrate this:

*I can feel that in this organization they only focus on the young people. Because you only see this digitization... We who are older then have a lot of experience that may not be quite as necessary today. It is more important that you are part of the digitization journey and embrace all this new technology and work on and so on*. (57-year-old woman, Sweden)

*There is this ingrained idea that they [older] are not flexible, they just did not get the knack of computers, do not know languages, which may be true, but more likely it is not true, but it is deeply ingrained*. (66-year-old man CZ)

Our analysis shows that older workers are suffering from a fear that they might be excluded in the ongoing digitalization process, a lack of techno-inclusion (Nimrod, 2018). We have shown that the informants in both countries perceive that their knowledge and long-term experience is not appreciated and that the stereotypical low evaluation of the older worker's digital competences is not a matter of a particular department or a single manager, but that it is deeply rooted and widespread in the organization. These experiences with digitalization and the consequences for the older staff in both countries can be interpreted as expressions of ageism, regardless of whether it is defined as prejudice and discrimination against older people (Wilkinson and Ferraro, 2002), or as the maintenance of asymmetrical power relations based on age (Krekula et al., 2018).

#### 5.2.3 Insecurity about the future

Our material shows that organizational changes leading to restructuring teams and banks as such in Czechia and Sweden make older workers feel insecure about their jobs. The following accounts of informants reveal their experiences of disappearing job security and the possibility of being made redundant:

*I was always very stressed by the idea of not having any security. I wanted to be rooted somewhere … and not be threatened by redundancies or cuts and I will not fear that I will suddenly lose my job. However, I don't have this security. Anything can happen here*. (53 years old, woman, CZ)

“*But you never know, the rug could be pulled away tomorrow.” And that's how it is... It has become part of everyday life that no one feels safe. But.. you can only relate: Today is good. And then I know nothing more*. (53 years old, woman, Sweden)

The data also show that the experience of feeling insecure about one's own future stems from the past experiences of their older colleagues who were made redundant or forced to leave, as described by this 55-years-old Czech female banker: “*I was hoping that I could stay until retirement here …. it was just a colleague of mine who, somehow, they made him leave five years before retirement*.” The following quotation from Sweden goes in a similar way:

*And a great woman in our office, who was almost like... forced to leave. Even though she didn't really want to. Because they wanted to replace her with someone younger. … it was very clear that they... tried to influence her to make this decision herself. … She is not the only one who has found herself in this situation*. (57 years old woman, Sweden)

The quotes above illustrate that one's own uncertainty about the future is based on how one has experienced colleagues being treated; the treatment they have received is what one expects for oneself. This can be seen as an expression of how experiences among colleagues at the workplace intertwine and contribute to the shaping of individuals' frameworks of interpretation, what is described in life course research with the term “linked lives” (Bengtson et al., 2005).

In addition to narratives about specific cases of colleagues being made redundant, our data also reveal more general narratives about older workers leaving and/or being made redundant. In both countries, informants come up with general narratives about previous reorganizations and waves of redundancies that mainly affected older workers:

*What stresses me out the most is when they start talking about having to reduce by one person again. So one simply begins to think: it will fall on me or it will not fall on me, whom will they choose?* (53 years old, woman, Czechia)

*But I don't feel that I can be 100% safe… we've seen that before. You can pull the carpet away and then you do it again. Or closing an office, we have also experienced that*. (60 years old woman, Sweden)

The above has illustrated that for the older employees in both countries, digitalization results in (techno) stress, a feeling of not being needed and in demand, and a perceived uncertainty about the future, primarily in the form of a fear of losing their job. Overall, this can be understood as an expression of a precarious work situation. The results align with Lazar and Sanchez (2019) pointing out that precarity is experienced also by employees who seemingly have a secure position in the form of contracts and stable wages. They also connect to Lorey (2020) argument that processes of precarization are widespread experiences that are part of the daily lives of many. The results specifically highlight the role that digitalization has for the older employees in these processes.

## 6 Discussion and conclusion

Based on qualitative interviews with finance employees aged 50 and older in Czechia and Sweden, this article illustrates how older employees experience digitalization and organizational changes.

Our findings illustrate that the introduction of agile management, is perceived as a central ongoing change in Czechia, while in the Swedish context, it emerges as an established working method. At the same time, outsourcing is described as a current change that significantly affects the studied Swedish bank branches, whereas it is not prominently mentioned in the Czech material. These differences can be understood as expressions of national variations. Digitalization, however, is described with remarkably similar narratives in both countries. This can be seen as an expression of the overall and extensive digitalization that has taken place in the finance sector globally. In other words, it illustrates that the assumption that digitalization creates increased efficiency necessary to obtain and maintain an international position is well established and widespread (Wajcman and Dodd, 2017).

Our results also show that the widely held assumption that digitalization will decrease workload and overall make work simpler does not correspond to experiences of older workers in any of the studied digitalizing workplaces. Instead, our results show that the restructuring of workplaces connected to digitalization is experienced as increased workload and increased tempo. Less people are expected to do more. In both Czechia and Sweden, the ongoing organizational changes are experienced as having rather negative consequences for older workers.

Overall, these findings indicate that there may be both similarities and differences in how narratives about organizational possibilities and challenges move within and between national work contexts. Analyses of organizational changes and their consequences for the older workforce therefore should not be based on homogeneous sector descriptions but should be grounded in analyses of national conditions and narratives. Our results indicate that older workers in Czech and Swedish banks experience digitalization as an organizational change similarly and with negative consequences. This may mean that digitalization is a process that is being introduced in a purely technical way and quite uniformly in workplaces in different countries, without considering the needs of employees, for example, according to age. Technology is often perceived as some kind of inevitable external force apart from social relations (Wajcman and Dodd, 2017). Therefore, also its negative consequences can be considered inevitable and given by technology itself, especially if specific workplace policies are not in place to mitigate the impact of technological change.

We have also illustrated that the organizational changes connected to digitalization create precarious working conditions for the older workforce in three ways: technostress based on rapid related organizational changes, anxiety and a sense of ineffectiveness (see Ayyagari et al., 2011), a feeling of not being needed and wanted, and uncertainty about the future. The technostress experienced by older workers is in our study a consequence of increased workload and work pace in contrast to expectations of work simplification and can be seen in close connection to the non-existence of systematic training and responsibilization of employees for their own training with new digital technologies introduced.

We have further argued that the narratives of not feeling valued in the workplace and the uncertainty about the future that it brings are also based on the informants' past experiences, including previous layoffs, and how they have seen older employees being treated within the organizational context. This shows that older workers' experiences of ageism are not based only on isolated cases of ageism toward their colleagues, in terms of linked lives, but also as schematic narratives existing within the organizations. These schematic narratives about events, such as budget cuts or abolishment of jobs or whole departments, are automatically raising expectations of dismissals or discriminatory treatment of older workers and therefore are reflecting the deeply embedded ageism within the organizations. Ageism in the form of not feeling needed and internalized (Hampel and Kunze, 2023), as not having the skills needed, has been illustrated specifically in relation to digitalization and within the banking sector organizations that are at the forefront of digitalization. Current understanding of the organizational culture as ageist is based on past own or colleagues' experiences as well as on schematic narratives embedded within the workplace. Older finance workers in both Czechia and Sweden projected these experiences on themselves and felt insecurity about their own future job prospects. Those past experiences and internalized ageism therefore resulted in precarization as experienced by older women and men.

These findings mean that the position of older workers in the digitalizing banking organizations is not very strong and that there is a culture of ageism, which eliminates older workers, either makes them directly redundant or makes them decide to leave due to negative impacts it has on their work experiences.

### 6.1 Limitations

The research presented has some limitations related to its qualitative methodology, as well as possibly some limitations in terms of its time validity. Due to the focus on two specific countries (Czechia and Sweden) and one specific sector (banking), the results cannot be generalized to all contexts and sectors. However, this focus enabled us to explore differences and similarities in the sector and context between the two countries, which differ greatly in terms of digitalization. This research approach enabled us to gain a more in-depth understanding of how the digitalization process can affect older workers, providing a nuanced view of its impact on extending careers in the two countries and one sector. Our analytical process aimed to reach saturation of the themes detected in the data. At one stage of the analysis, therefore, we decided to conduct additional interviews in Czechia to explore themes related to organizational changes in more depth. Although most of the data were collected in 2019–2020, we conducted additional four expert interviews in 2023.

This brings us to the second area of limitations. Most of the data was collected several years ago, just before the onset of the COVID-19 pandemic, a period during which the digitalization of work intensified in most sectors. Despite the time gap between data collection and publication of the results, we believe that they are still valid. Even before we started collecting our data, banking was at the forefront of digitalization among other sectors, and we entered a field in which organizational changes were ongoing, albeit at different stages in the two countries, as we demonstrate. Therefore, we believe that our findings are also valid for sectors that started digitalising later than banking, or that are implementing other technological changes or workplace disruptions. The experiences of older workers that we analyse are not specific to the time when our data was collected; they could be applicable to digitalised workplaces or workplaces introducing new technological processes and organizational changes, at least as a hypothesis for further research. Furthermore, there is a significant research gap in the literature focusing on how older workers experience organizational changes connected to technological and policy changes that extend their working lives. We believe that our research can motivate further research and comparisons with our findings, as well as providing valuable policy implications and recommendations.

### 6.2 Implications, policy recommendations, and further research

Our research shows that, despite calls from the OECD (2019) and other bodies for workplace measures to support workers in extending their working lives, workplaces are not prepared for the implementation of new technologies. In a constantly changing organizational landscape involving software and other technologies, workplaces should pay more attention to training that corresponds to workers' needs and avoid holding workers responsible for their own training. Workplaces that do not support workers in adapting to new technologies and are restructuring, risk that age becomes the basis of inequality, making workers feel excluded and in a precarious position. Further, training should be provided to middle and line management about bias, including age bias, to increase their ability to create a non-discriminatory workplace culture. Although our sample and analysis did not detect differences based on gender, race, ethnicity or migration status, further research should investigate how other lines of inequality and their intersections impact the experiences of workers in digitalised workplaces.

Furthermore, as digitalization can have a positive or negative impact on the ability to work longer, and further research could examine how digitalization can support the EWL in different contexts. For instance, remote working could be promoted, and further research might identify, where it is possible and convenient (Peng et al., 2017). The key factors seem to be how digitalization is implemented and the level of support provided, considering country-specific and sectoral contexts. Research shows that the priorities and strategies of the organizations that own and operate the technology are important in its implementation process (Howcroft and Taylor, 2023). Therefore, the role of various stakeholders, such as trade unions, should also be considered, but it has not been sufficiently researched. Digitalization undermines the EWL when proper workplace training is not provided, and when digital technology is associated with negative age stereotypes. Organizations should implement programmes to support the EWL, including, for example, relocations to other jobs within the workplace or organization. Also, evaluations of these policies, where they have already been implemented, would be needed for further research.

Our research also showed that temporality is important, as older workers' agency emerges at the intersection of past experiences and future expectations (Keskinen et al., 2023). Therefore, our results highlight the value of adopting temporal analyses, although we did not have the capacity to develop this topic further in this manuscript. Further research could focus on the temporal dimension of organizational workplace dynamics and explore how current understandings relate to the collective past.

## Data Availability

The data analyzed in this study is subject to the following licenses/restrictions: Due to privacy reasons the data is only available on request. Requests to access these datasets should be directed to alena.krizkova@soc.cas.cz.

## Footnotes

1 https://csu.gov.cz/ (Accessed February 15, 2026).

2 https://www.scb.se/en/ (Accessed February 15, 2026).

## Supplementary material

Supplementary file 1 Interview guide for employees.

Supplementary file 2 Interview guide for experts.

Supplementary file 3 Coding structure.
