# Supplementary material for: The impact of digitalization and organizational changes on older workers' insecurity in the finance sector in Sweden and Czechia
Source: Front Sociol. 2026 Jun 25;11:1835265. doi: 10.3389/fsoc.2026.1835265 (PMC13345652; doi:10.3389/fsoc.2026.1835265)
Supplement: Supplementary file 1 — Interview guide for employees. [file Data_Sheet_1.PDF]

Dynamics of Accumulated Inequalities for Seniors in Employment (DAISIE) research project, funded under the NORFACE DIAL programme

Interview Guide – employees 50+

Current Job & Employment History

Elicit as much information as possible about **current job** & working conditions:

Length of service with the company

Reasons for choosing this particular job / company (reputation, lack of choice, dissatisfaction in previous job, etc.)

Recruitment procedures (formal / informal / personal network)

Number of hours worked and patterns (shift work, etc.) + changes over time

Job content (e.g. describe a typical working day / what you did last Monday)

Latitude in organising working time and job content (flexibility, autonomy, control)

Most / least enjoyable aspect of work?

- Prompt around issues of stress, physical exertion, dealing with clients, relationships with colleagues, being recognised and rewarded, the (growing) digitalisation of work (use of internet, social media), etc.

Appreciation of current job in comparison to previous employment experiences?

- Link to **employment history**

Work-history from 1st job up until current job

Links to family & health events (life-grid)

Real or Expected Effects of (one's own) Ageing on Work

Changes in own attitude towards work (in general), current job or employer over time?

Perceived changes in working conditions (increased pressure, intensity, work overload *versus* routinisation, experience, technical abilities) over time?

Perception of changes in attitudes towards self or older workers in general on the part of colleagues, clients, bosses over time?

Perception of ability to carry out duties until / beyond (expected) retirement age

- Health considerations (cf. life-line)
- Organisational reforms / changes / issues on the horizon (incl. digitalization)
- Special conditions for older workers (e.g. extended paid holiday rights)
- Family events, incl. actual or expected child / elder-care duties (cf. life-line)

Organisational Attitudes towards Older Workers

Discussion around retirement transition / extending working life within the company?

Perception of company as supportive of older workers (+ women / minorities) or not?

- Retirement preparation training (Timing? Useful? Accessible?)
- Health & safety initiatives (Well-being modules? Complimentary health insurance provision?)
- Adaptation of work station / job content (Evaluation procedures?)
- Understanding of / arrangements around potential care commitments (formal or via line-manager / N+1)
- Opportunities for retraining / up-dating knowledge / adapting to new technologies / new work practices (systematic or only on request)
- Signs of stigmatization / marginalization of older workers?
- Union involvement in ageing / equality / anti-discrimination issues?
- Knowledge of family-friendly measures potentially available, if needed.

Examples of colleagues who were encouraged to leave / stay until / beyond 65 years.

Examples of adjustments for people returning from sick-leave / caring for a family member.

Experienced or observed age and/or gender discrimination in this company?

Interactions with Family / Care Responsibilities

Current living arrangements & changes to household composition over time (incl. home ownership, mortgage arrangements, residential mobility).

Domestic labour arrangements: Share of household duties at the moment? Changes over time? Effect on household employment patterns now / in the past?

Nature and regularity of personal (or partner's) involvement with provision of (care) services to: (step-)children / grandchildren; elderly parents [in-laws] / grandparents / other family members / neighbours / friends, etc.

Implications of employment / working patterns on caring aspirations / duties (and vice versa)

Attitudes towards caring duties? If you had the opportunity, would like to increase / decrease / maintain the same level of care provision (including domestic labour)?

What prevents / facilitates doing so?

Expectations about future caring roles? Residential proximity of extended family members?

Health implications of caring duties? Satisfaction / frustration / exhaustion?

Transition to Retirement

Already started thinking about retirement? Concrete retirement plans?

When do you expect to retire?

What about your partner's retirement plans?

Expectations for retirement calendar (ideal versus expected retirement pattern)

Main factors taken into consideration when deciding to retire, partially retire, or remain in employment. How important are these things and why?

- Statutory regulations (country / sector / company-specific?)
- Household finances (prompt on contribution to overall household income)
- Partner's situation (age difference, employment history, health, hobbies, etc.)
- Family care needs (link to employment patterns / aspirations of daughters [in law] and alternative childcare provision, including children's in-laws)
- Travel plans (including return migration)
- Health issues (link to possible adjustments to working time / job content)
- Other (e.g. hobbies, voluntary work, shared activities with spouse).

Financial circumstances

Can you tell me about your personal and/or household pension provision?

How will your income in retirement be funded? State pension? Occupational pension? Private pension? Pension splitting? Personal savings?

Do you own your own home, have a mortgage or rent your home?

Do you (or your partner) have other assets? Savings, property, etc.?

Were you aware of the need to plan/provide for your pension at the start of your working life? If not, when did you become aware?

Comparison with the retirement conditions of parents / siblings / friends / others: better / worse / difficult to judge.

Use laminated cards to elicit information about household income + pension provision.

Personal opinion on EWL issues

Before we bring this conversation to a close, I'd like to know what you think about the idea of encouraging people to work longer / to increase the [female] state pension age / to encourage people who have retired to combine their pension with income from a job? Have you thought about these issues or discussed them with anyone?

And finally, if you had to draw the pattern of your life, how would it look? And if you had the opportunity to plan your life over again, would you do anything differently?

Rounding up phase

I think that we have just about covered all the issues I wanted to discuss with you today. I would like to thank you for your time. It was very useful for me to get your point of view and life-history. Have we overlooked anything important? Is there anything you would like to ask me?

If you have any questions that spring to mind later, please don't hesitate to contact me [**Interviewer contact details**].

We have prepared a document about organisations that can provide information about the transition to retirement or extending working life. Would you like to receive a copy? [**Information sheet**].

dial Dynamics of Inequality Across the Lifecourse: structures and process
NORFACE ERA-NET
