# Supplementary material for: The impact of digitalization and organizational changes on older workers' insecurity in the finance sector in Sweden and Czechia
Source: Front Sociol. 2026 Jun 25;11:1835265. doi: 10.3389/fsoc.2026.1835265 (PMC13345652; doi:10.3389/fsoc.2026.1835265)
Supplement: Supplementary file 2 — Interview guide for experts. [file Data_Sheet_2.PDF]

Dynamics of Accumulated Inequalities for Seniors in Employment (DAISIE) research project, funded under the NORFACE DIAL programme

Expert Interview Guide

Thank you for agreeing to meet with me today. As you know, we are conducting a study funded by the European Union on the experiences of older workers in a number of employment sectors, including your own [**Project Presentation Document**].

In addition to carrying out interviews with older workers, about their experiences and plans for the future, we're also meeting with a number of experts, like yourself, people who in one capacity of another may be confronted with the issues of ageing at work or who may be dealing with extending working life challenges.

I have prepared a number of questions for you, but if there is anything that you feel are important to discuss and that I haven't covered, please feel free to mention this yourself.

Ageing Awareness within the Organisation

To begin with, can you tell me whether the issue of older workers / ageing at work is something that your organisation is confronted with at the moment?

If so, in what way? If not, why is that the case?

Can you describe the age profile of the work-force in your organisation?

Do you have any figures? Is this something that you monitor regularly? [Ask for a copy of any available reports / publications / data]

Is age something that you consider when recruiting new members of staff?

Has there been any discussion within your organisation about the idea of encouraging people to work longer?

What do you think about the potential increase to the state pension age? Will this pose any particular problems within your organisation? Have you started to plan for this?

What about the idea of encouraging people who have retired to combine their pension with income from a job?

Do you envisage any of these issues becoming more important for your organisation in the future?

Organisational Attitudes towards Older Workers

Would you describe your organisation as being supportive of older workers?

Do you provide special conditions for older workers (e.g. extended paid holiday rights)?

Has your organisation introduced any of the following measures? [If not, why not?]:

- Retirement preparation training (Eligibility conditions?)
- Health & safety initiatives (Well-being modules? Complimentary health insurance provision?)
- Adaptation of work station / job content (Evaluation procedures?)
- Arrangements around care commitments (Formal or informal?)
- Opportunities for retraining / up-dating knowledge / adapting to new technologies / new work practices (systematic or only on request?)

Are these age management measures related in any way to gender / ethnic discrimination policies and practices?

Have you ever witnessed signs of stigmatization / marginalization of older workers in this organisation?

Is your organisation involved in discussions around ageing / equality / anti-discrimination issues, for example with:

- trade union organisations?
- voluntary organisations?
- local government bodies?

Examples of staff who were encouraged to leave / stay until / beyond 65 years?

Examples of adjustments to working conditions for older people returning from sick-leave / caring for a family member?

Examples of age discrimination issues within the organisation, in the past or recently?

Personal Attitudes towards Ageing at Work

Perhaps we could turn to your personal situation now and the way you imagine your future transition to retirement **[aim = to personalize the account in order to avoid being given the official company discourse on EWL, with no empirical basis]**

[*It's probably still a long way off, but…]* Have you already started thinking about your own retirement plans?

Do you expect to still be working for this organisation by then? And to be doing the same kind of job?

Do you expect to be able to carry out your current duties / level of engagement until / beyond (expected) retirement age? Why?

What kind of advice or support would you expect or like to receive from your company as you move towards retirement age?

Rounding up phase

I think that we have just about covered all the issues I wanted to discuss with you today. I would like to thank you again for your time. It was very useful for me to get your point of view and to hear more about your organization.

Have we overlooked anything important?

Is there anyone else in your organisation that we should be talking to?

Is there anything you would like to ask me?

If you have any questions that spring to mind later, please don't hesitate to contact me [**Interviewer contact details**].

As you know, we are looking to carry out interviews with older workers (50+) in a range of employment sectors, in order to gauge the issues they face as they get older at work.

Would you be willing to help us set up an interview panel within your organisation? [**Company invitation letter**].

Dynamics of Inequality Across the Lifecourse: structures and process
NORFACE ERA-NET
