# Supplementary material for: The impact of digitalization and organizational changes on older workers' insecurity in the finance sector in Sweden and Czechia
Source: Front Sociol. 2026 Jun 25;11:1835265. doi: 10.3389/fsoc.2026.1835265 (PMC13345652; doi:10.3389/fsoc.2026.1835265)
Supplement: Supplementary file 3 — Coding structure. [file Data_Sheet_3.PDF]

Thematic coding scheme		
Organizational changes	Agile management	Still learning, implementation process<br>Established routine<br>Accepted practice
	Outsourcing	Marginal process<br>Major process<br>Insourcing
	Digitalization	Myth of symplifying + reducing work<br>Workload increase<br>Workpace increase<br>Responsibility for training
Experiences of insecurity	Stress	Increased stress (example – standing presentation)<br>Changing software<br>Increased tempo<br>New regulation – administration increase<br>Technostress
	Ageism – older workers don´t feel needed and wanted	Having adequate knowledge<br>Outdated skills<br>Exclusion
	Insecurity about the future	Possibility of redundancy<br>Experiences with colleagues made redundant<br>Precarious work situation
